# Insect anal droplets contain diverse proteins related to gut homeostasis

**DOI:** 10.1186/s12864-018-5182-z

**Published:** 2018-10-30

**Authors:** Tianzhong Jing, Fuxiao Wang, Fenghui Qi, Zhiying Wang

**Affiliations:** 1 School of Forestry, Northeast Forestry University, Harbin, 150040 China; 2 School of Life Sciences, Northeast Forestry University, Harbin, 150040 China

**Keywords:** Anal droplet, Intestine, Innate immunity, Phagocytosis, *Cryptorhynchus lapathi*

## Abstract

**Background:**

Insects share similar fundamental molecular principles with mammals in innate immunity. For modulating normal gut microbiota, insects produce phenoloxidase (PO), which is absent in all vertebrates, and reactive nitrogen species (ROS) and antimicrobial proteins (AMPs). However, reports on insect gut phagocytosis are very few. Furthermore, most previous studies measure gene expression at the transcription level. In this study, we provided proteomic evidence on gut modulation of normal microorganisms by investigating the anal droplets from a weevil, *Cryptorhynchus lapathi*.

**Results:**

The results showed that the anal droplets contained diverse proteins related to physical barriers, epithelium renewal, pattern recognition, phenoloxidase activation, oxidative defense and phagocytosis, but AMPs were not detected. According to annotations, Scarb1, integrin βν, Dscam, spondin or Thbs2s might mediate phagocytosis. As a possible integrin βν pathway, βν activates Rho by an unknown mechanism, and Rho induces accumulation of mDia, which then promotes actin polymerization.

**Conclusions:**

Our results well demonstrated that insect anal droplets can be used as materials to investigate the defense of a host to gut microorganisms and supported to the hypothesis that gut phagocytosis occurs in insects.

**Electronic supplementary material:**

The online version of this article (10.1186/s12864-018-5182-z) contains supplementary material, which is available to authorized users.

## Background

Essentially, gut microbiota are composed of both commensal and pathogenic microbes, and they may be benign, beneficial or pathogenic in basal conditions [1, 2]. For multicellular organisms, nonself recognition is particularly important to protect an individual from potential environmental pathogens. Additionally, it is now firmly established that the animal gut immune system not only eliminates microbial pathogens but also maintains an adequate level of commensal microbiota [1, 3, 4].

The molecular basis of the interactions between the host gut epithelium and microorganisms has been investigated extensively in mammals and *Drosophila*. Mammal intestinal epithelial cells (IECs) keep the microbes at bay by secreting highly glycosylated mucins, reactive oxygen species (ROS) and antimicrobial proteins (AMPs) and by establishing a physical and biochemical barrier to microbial contact with the epithelial surface and underlying immune cells [4]. Specialized mammal IECs, called microfold cells (M cells), are capable of both specific receptor-mediated microbial uptake and nonspecific antigen uptake from the intestinal lumen [4]. Insects and other invertebrates share similar fundamental molecular principles with mammals in innate immunity [5]. In addition to ROS and AMPs, invertebrates also produce phenoloxidase (PO), which is absent in all vertebrates [3, 6]. Many insect guts also have a peritrophic envelope preventing microorganisms from direct contact with the epithelial cells [6]. In contrast to many reports on gut AMPs and ROS, reports on insect gut phagocytosis are very few. Whether any immune cells are associated with the *Drosophila* intestine remains an open question [7], although cells able to phagocytose bacteria and dying cells are present in the *Drosophila* proventriculus (PV) [8].

To study insect gut immunity, most previous studies measure gene expression at the transcriptional level [9]. In this study, we provided proteomics data from the anal droplets of a weevil, *Cryptorhynchus lapathi* (L.) (Coleoptera: Curculionidae). Insect feces are anal excretions either in liquid form or packaged in pellets and known as frass. Malpighian tubules initiate the insect excretory process. The primary urine from the Malpighian tubules mixed with the end products of digestion from the midgut are modified by continued resorption by rectal glands to produce a secondary urine that is then expelled through the anus. The liquid form of insect feces is called honeydew, anal secretion or anal droplet. Most insects produce either frass or anal droplets, whereas few insects produce both. The poplar-and-willow borer, *C. lapathi*, is a wood-boring pest of economic importance throughout Europe, China, Japan, the United States and Canada [10]. When disturbed, the larvae produce anal droplets. As the larvae spend all their life within a tree stem, this phenomenon has never been previously reported.

Previous studies revealed the chemical composition of hemipteran honeydew, which primarily contains sugar, amino acids and other chemicals [11]. Unfortunately, the proteins in insect anal droplets were not reported until small AMPs were detected in the anal secretions of *Nicrophorus* (burying beetles) [12]. Additionally, very recently, proteomic investigation of aphid honeydew revealed an unexpected diversity of proteins [11]. Thus, the gut immune system can possibly be reconstructed by investigation of an anal droplet proteome.

In this paper, a proteome investigation of the larval weevil showed many proteins related to physical barriers, epithelium renewal, pattern recognition, phenoloxidase activation, oxidative defense and phagocytosis, but no AMPs were detected.

## Materials and methods

### Anal droplet collection

Larval weevils were collected in the wild. Anal droplets were collected directly onto foils placed against the anal areas of each weevil, while gently squeezing their abdomens. Samples on the foil were then moved into a mini glass bottle with a pipette and stored at − 20 °C when not immediately used.

### Q-TOF MS sequencing

**Fig. 1:**
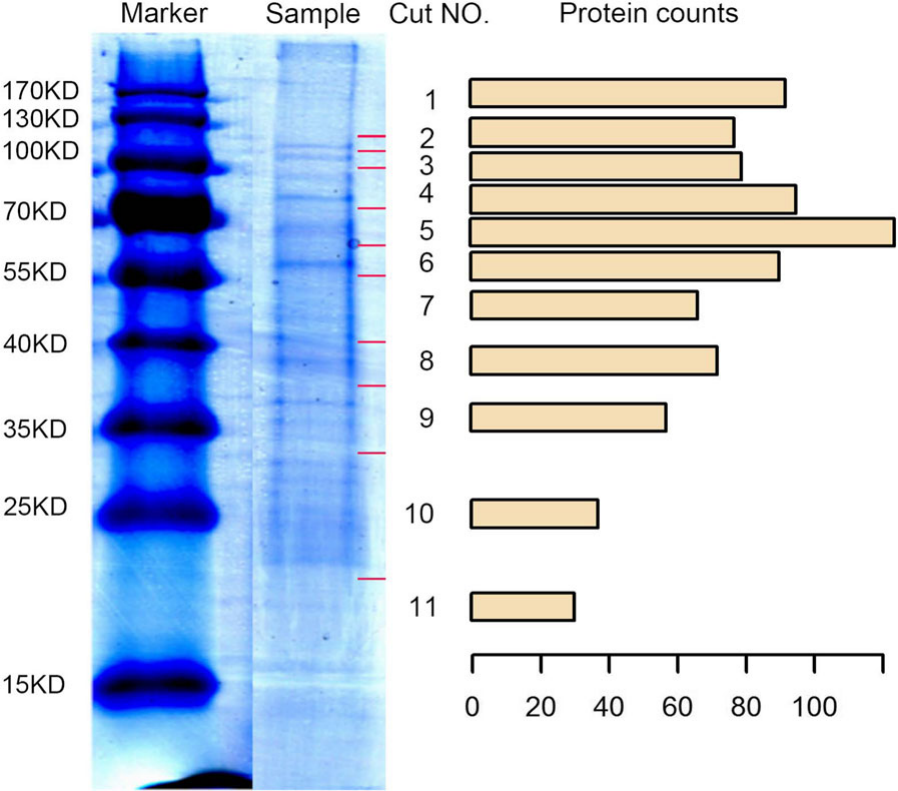
SDS-PAGE of the anal droplets. The gel was cut into 11 segments at the red line for Q-TOF. The counts of identified protein from each segment are shown in a barplot

The anal droplets were first separated on SDS-PAGE before LC-MS analysis. Ten microliters of pooled sample was resuspended in 50 μL of Laemmli sample buffer supplemented with 2% β-mercaptoethanol and heated at 95 °C for 5 min. After electrophoresis, the gel was rinsed with three changes of Nanopure water, stained for 20 min with Bio-safe TM Coomassie and destained with three changes of Nanopure water. The gel lane was carefully cut into eleven pieces (Fig. 1), placed into Eppendorf tubes and rinsed twice for 10 min with 1 mL of MilliQ water. After destained with freshly prepared destaining solution (25 mM (NH_4_)HCO_3_, 50% acetonitrile), the gel pieces were dehydrated until they shrank and became white (approximately 2 min) with 25 mM (NH_4_)HCO_3_ with 50% acetonitrile and then once more for 30 s in 100% acetonitrile. The gel pieces were then rehydrated in freshly prepared 10 mM Dithiothreitol for 1 h at 56 °C (water bath) and were alkylated with freshly prepared 55 mM Iodoacetamide for 1 h at room temperature in the dark. Subsequently, the gel pieces were washed with 25 mM (NH_4_)HCO_3_ twice for 10 min and destained to become white as before. Trypsin digestion was performed overnight with trypsin work solution (1 μg/uL stock solution was diluted 15-fold with 25 mM (NH_4_)HCO_3_) at 37 °C. Digested proteins were extracted 4 times with 50 μL of 50 mM (NH_4_)HCO_3_, 50 μL of 0.1%(*V*/V)FA in water, 50 μL of 0.1%(V/V)FA in acetonitrile and 50 μL of acetonitrile. All extracts were pooled, freeze-dried at − 20 °C and resuspended in 0.1% FA for sequencing.

The resuspended peptides were fractionated using reversed-phase high-pressure liquid chromatography (HPLC; prominence nano 2D, Shimazu, Kyoto, Japan), and the gradient-eluted peptides were analyzed using a MicrOTOF-QII (Bruker Daltonics, Billerica, MA, USA). The liquid chromatography columns were packed in-house with C18 (5 μm, 150 Å; Downers Grove, IL, USA). LC-MS conditions were as follow: Mobile phase: (A) 100% H_2_O with 0.1% FA and (B) 100% acetonitrile with 0.1% formic acid; Gradient: 0–4 min, 5–5% B; 4–30 min, 5–40% B; 30–35 min, 40–80% B; 35–45 min, 80–80% B; 45–45.1 min, 80–5% B; 45.1–60 min, 5–5%; Flow rate, 400 nL/min; Drying gas temperature, 150 °C; Capillary voltage, 1.5 kV; Collision gas, argon. The results were exported as. MGF file for X!Tandem [13] analysis.

### Database searching and protein identification

For protein identification, the peak list data from MS were searched against a protein database. Instead of downloading insect proteins from a public database (for example, National Center for Biotechnology Information (NCBI)), we constructed a protein database of *C. lapathi* itself. A transcriptomic database of *C. lapathi* was first constructed by de novo assembly (Trinity software [14]) of the sequences from an Illumina sequencing platform (Illumina HiSeq2500) based on pooled RNAs of the larvae, pupae and adults and was then clustered by CD-HIT software (http://weizhongli-lab.org) to obtain unigenes. The unigenes were subsequently mapped to the proteome of *Dendroctonus ponderosae* with a cutoff E-value of 10^− 4^ using BLASTx to obtain a proteomic database of *C. lapathi*. The proteome of *D. ponderosae* was downloaded from UniProtKB (http://www.uniprot.org/). The database searches were performed by an R package, rTANDEM [13]. The proteins were identified from at least one peptide and with an X!tandem [15] score corresponding to an expected value of better than 0.05.

We performed BLASTP against the Antimicrobial Peptide Database (APD) [16] to identify AMPs from the anal droplet. For identification of other enzymes, local databases were set up and BLASTP was conducted.

### Assignment of proteins to Kyoto encyclopedia of genes and genomes (KEGG) pathways

The IDs of proteins identified by X!tandem were labeled with their homologue gene IDs (UniProtKB protein entry, http://www.uniprot.org) of *D. ponderosae*, and then KEGG IDs were obtained from UniProtKB using the ID Mapping function. Enrichment analyses of KEGG were conducted by an R package, GOstats [17].

## Results

### Proteins identified by LC-MS

The BLASTX results showed that 26,685 unigenes of *C. lapathi* were aligned to the proteome of *D. ponderosae*. The deduced amino acid sequences of these unigenes were used for the database search by X!tandem. In total, 819 proteins homologous to *D. ponderosae* were identified (Fig. 1), and those related to gut homeostasis are summarized in Table 1.

**Table 1:** Identified proteins with putative defensive roles from anal droplets of *Cryptorhynchus lapathi*

Putative Roles	Protein names	Homologous proteins of *Dendroctonus ponderosae*
Physical barrier	mucin; ZO-1	XP_019757616.1; XP_019771453.1
Wnt signaling pathway	CTNNB1; RUVBL1; WNT7; SIAH1; AXIN1; PLCB; CSNK2A	XP_019758083.1; XP_019762013.1; XP_019763573.1; XP_019765030.1; XP_019766713.1; XP_019770066.1; XP_019772815.1
Pattern recognition	PGRP-LB; Lysmd2; TLP; apoLp; Dscam; Comp; spondin2; Peroxinectin; SCARB1; integrin; C-Lectin; C-Lectin; GNBP	XP_019769175.1; XP_019755182.1; XP_019760168.1; XP_019773452.1; XP_019762301.1; XP_019755181.1; XP_019768788.1; XP_019767755.1; XP_019762441.1; XP_019770383.1; XP_019766712.1; XP_019769040.1; XP_019759712.1
Phenoloxidase activation	PPO2; iPLA2-gamma; serpin24; H2; P27; P40; P127; P80; trypsin 3A1-like; P128; trypsin-7-like; VSP34; VSP-like; SP K12H4.7; H129; Spn88Ea; P155; P127; H146	XP_019754719.1; XP_019769822.1; XP_019767866.1; XP_019756791.1; ERL89372.1; XP_019771903.1; XP_019768426.1; XP_019772019.1; XP_019767905.1; AEE62374.1; XP_019773738.1; XP_019768627.1; XP_019764390.1; XP_019768322.1; XP_019753952.1; XP_019767187.1; XP_019773128.1; AEE63209.1; XP_019767340.1
Oxidative defence	ferritin; transferrin; SOD; PRX; PRX; CAT; NOX5; SOD; Vg	XP_019756020.1; XP_019766109.1; XP_019766433.1; AEE61832.1; XP_019771831.1; XP_019760743.1; XP_019771050.1; XP_019769059.1; XP_019765469.1
AMPs	none	
IMD signaling pathway	Caspar; IMD; TAB2; TAB1	XP_019764393.1; XP_019765010.1; XP_019761007.1; XP_019771893.1
Toll signaling pathway	persephone; pelle; TLR7; spz4; cactus; Rnf41; toll; cactin	XP_019770363.1; XP_019760028.1; XP_019754070.1; XP_019771425.1; XP_019761667.1; XP_019760096.1; XP_019761038.1; XP_019769310.1
Lysosome pathway	uidA; NAGA; uidA; CTSL; CTSB; MAN2B1; LIPA; CTSL; GGA; MAN2B1; E3.2.1.25; SMPD1	XP_019754382.1; XP_019754675.1; XP_019757545.1; XP_019757796.1; XP_019761602.1; XP_019766480.1; XP_019767488.1; XP_019770434.1; AEE61763.1; XP_019771763.1; XP_019772256.1; XP_019772384.1
Endocytosis	HSPA1s; HGS; RNF41; HSPA1s; IQSEC; HSPA1s; SH3GL; RAB11FIP1_2_5; DAB2; HSPA1s; EHD1; PSD; RABEP1	XP_019755343.1; XP_019758450.1; XP_019760096.1; XP_019760386.1; XP_019760403.1; XP_019760659.1; AEE62651.1; XP_019760771.1; XP_019765026.1; XP_019765222.1; XP_019767475.1; XP_019770187.1; XP_019772712.1
Phagosome	ACTB_G1; ACTB_G1; CTSL; SEC22; ITGB1; CTSL; ACTB_G1; THBS2S; ACTB_G1; HGS; ATPeV1C	XP_019754675.1; XP_019755181.1; XP_019757620.1; XP_019758489.1; XP_019758493.1;; XP_019760672.1; XP_019765810.1; XP_019769732.1; XP_019770187.1; XP_019770383.1; XP_019772384.1
Phagosome formation	actin; cofilin; SEC22BB; actin; formin; ILK; actin (cytoplasmic); Aip1; Carmil1	XP_019769732.1; XP_019760098.1; XP_019765810.1; XP_019760672.1; XP_019765699.1; XP_019770853.1; XP_019758489.1; XP_019761028.1; XP_019762739.1
Phagosome maturation	Rab-3; V-ATPase; Rab32; cathepsin L; Rab14; Rab27A; vps54; kif23; cathepsin B; kif18A; dynein; milt; kinesin 2; cathepsin L1	XP_019769835.1; XP_019757620.1; XP_019753634.1; XP_019772384.1; XP_019761359.1; XP_019755005.1; XP_019762224.1; XP_019759104.1; XP_019757545.1; XP_019759859.1; XP_019756852.1; XP_019771654.1; XP_019761295.1; XP_019754675.1
Phagocytosis regulation	Arhgap39; Tbc1d9; Sgsm3; Srgap1; DOCK9; Tbc1d13; abr; Arhgef5	XP_019753574.1; XP_019763036.1; XP_019765184.1; XP_019765347.1; XP_019761336.1; XP_019773504.1; XP_019766889.1; XP_019771636.1
Defense against virus	argonaute; Dicer	XP_019768859.1; XP_019773735.1
Other	chitinase; chitinase; chitinase; chitinase; Etl1 (CG5899)	XP_019756484.1; XP_019767256.1; XP_019756184.1; XP_019757751.1; XP_019761024.1

### Proteins functioning as a physical barrier

**Fig. 2:**
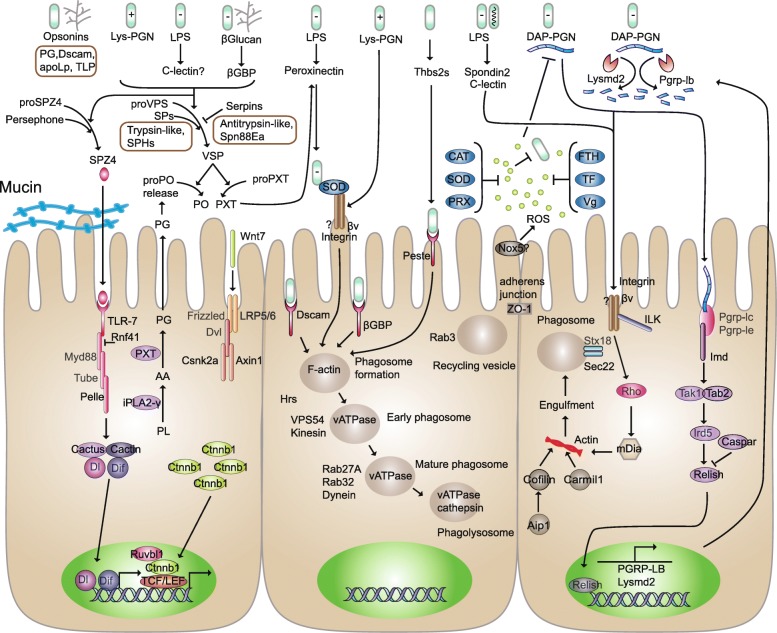
Anal droplet-derived proteins and possible pathways in innate immune. Proteins of which labels in grey were not detected in the anal droplets. ApoLp, apolipophorins; CAT, catalase; CTLs, C-type lectins; DAP, *meso*-diaminopimelic acid; FTH ferritin; LPS, lipopolysaccharide; PGN, Peptidoglycan; PGRPs, peptidoglycan recognition proteins; PO, phenoloxidase; proPO, prophenoloxidase; PRX, peroxiredoxin; PXT, peroxinectin; RNS, Reactive nitrogen species; ROS, reactive oxygen species; SOD, superoxide dismutase; TF, transferrin; TLPs, Thaumatin-like proteins; Vg, Vitellogenin; VSP, venom serine protease; ZO-1, Zonula occludens-1; βGBP, beta-1,3-glucan-binding protein

Two proteins, mucin and ZO-1 (Zonula occludens-1), were identified from the anal droplet (Additional file 1: Table S1; Fig. 2). Insects have a mucus layer that lines enterocytes along the midgut, and the production of mucin is regulated by infection [1]. Tight junctions connect adjacent IECs and could be important targets for increasing the integrity of the intestinal barrier [4]. Occluding junctions play crucial roles in epithelial barrier function. The occluding junctions found in vertebrates are called tight junctions; whereas invertebrate species have a different type of occluding junction, the septate junction (SJ). Arthropods contain two types of SJs, the pleated SJ (pSJ) and the smooth SJ (sSJ). The pSJ exists in ectodermally derived epithelial cells and glial cells, whereas the sSJ is found in endodermally derived epithelia, such as in the midgut of insects. Malpighian tubules of insects also have sSJs. Together, Tsp2A (tetraspanin 2A), Ssk (snakeskin) and Mesh form a protein complex, and all three proteins are required for sSJ formation and for intestinal barrier function in *Drosophila* [18]. From the anal droplet, none of these proteins were detected; whereas a tight junction–associated protein, ZO-1, was detected (Additional file 1: Table S1). ZO-1 and other homologues of vertebrate tight junction proteins do not localize to the septate junction but instead are found in adherens junctions or the marginal zone (reviewed in [19]). In insect epithelial cells, adherens junctions are at the apical apex and septate junctions localize more to the basal domains; therefore, ZO-1 perhaps plays the role of physical barrier similar to that in humans and mammals. In humans, normal gut microbes induce the expression and redistribution of ZO, suggesting the weevil gut microbes also regulate adherens junctions [20].

### Proteins involved in gut epithelium renewal

Gut epithelium renewal is considered a tolerance mechanism of insects to increase the capacity to endure infection. This response depends on induction of the Wingless, JAK-STAT and Epidermal growth factor receptor (Egfr) pathways in progenitor cells, which stimulates their proliferation and increases their differentiation into enterocytes (reviewed in [1]). Egfr ligands are required to activate the Egfr pathway. *Drosophila* Egfr and its activity are modulated by five ligands. The TGF-α-like molecules, Gurken (Grk), Spitz (Spi), and Keren, and the neuregulin-like molecule Vein (Vn) function as receptor activators. The fifth Egfr ligand, Argos (Aos), is a receptor antagonist (reviewed in [21]). We did not find any coleopteran Grk homologs by BLASTP against the Nr database, whereas the other four Egfs were found in the weevil transcriptome. However, none of them were detected from the anal droplet. From the anal droplet, only one enzyme (Sos) for JAK-STAT pathways was identified, suggesting that the JAK-STAT pathway was not activated in the basal condition in gut epithelial cells. By contrast, seven proteins involved in the Wnt signaling pathway were identified, which were Ctnnb1, Ruvbl1, Wnt7, Siah1, Axin1, Plcb and Csnk2a (Additional file 2: Table S2; Fig. 2), suggesting a canonical Wnt pathway was activated. These results are consistent with the conclusion that the gut microbiota also promote epithelial renewal in basal conditions [1].

### Proteins involved in pattern recognition

When microbial lipopolysaccharides (LPSs), glucans, or peptidoglycans are present, the coagulation cascade is triggered and/or changes to Ca^2+^ concentration or pH occur that activate a series of serine proteases to activate prophenoloxidase (proPO) in the hemolymph [22]. From the anal droplet, one beta-1,3-glucan-binding protein (βGBP, also known as GNBP), two C-type lectins (CTLs), one spondin 2 and one peroxinectin were detected (Additional file 1: Table S1; Fig. 2). βGBP binds to beta-1,3-glucan to initiate activation of cell-free prophenoloxidase [23], phagocytosis or Toll signaling [24]. CTLs are involved in opsonization, nodule formation, agglutination, encapsulation, melanization, and prophenoloxidase activation, in addition to maintaining gut microbiome homeostasis [25]. Binding to mannose, fucose, and glucose, among others, C-type lectin binds to β-integrin to promote hemocyte phagocytosis in an invertebrate [26, 27]. C-type lectins are classified into seventeen subgroups. It is difficult to classify the two C-type lectins from the anal droplet because of the very low similarity to the known CTLs. The extracellular matrix protein mindin (spondin) is a pattern-recognition molecule binding LPSs and an integrin ligand and is critical for initiating innate immune responses to both bacterial and viral pathogens (reviewed in [28]). Peroxinectin is a cell adhesive protein functioning in degranulation, encapsulation enhancement and with opsonin and peroxidase (reviewed in [29]). Insect phagocytic receptors include scavenger receptors, the nimrod receptor superfamily, peptidoglycan-recognition receptors, integrins, Dscam, and complement-like molecules [30] and can be divided into nonopsonic or opsonic receptors [31]. From the anal droplet, neither nimrod receptor superfamily receptors nor TEPs were identified, whereas one scavenger receptor class B member 1 (Scarb1, also known as peste in *Drosophila*), one integrin (beta-nu-like, also known as βν, betaInt-nu, βInt-ν, Βν, Dmel\CG1762, Fly betanu, Itgbetanu), one Dscam, one spondin-2 (also known as mindin) and one cartilage oligomeric matrix protein (Comp, also known as Thbs2s, thrombospondin-5) were detected in the anal droplet (Additional file 1: Table S1; Fig. 2). SR-I1 binds to both gram-negative and gram-positive bacteria [32] and is required for the efficient phagocytosis of two intracellular bacterial pathogens in *Drosophila* [33]. Dscam contributes to phagocytosis in *Drosophila*, mosquito and crayfish [34] and is considered a phagocytic receptor or opsonin for bacteria, which is conserved in invertebrates [30]. Integrin beta-nu transcripts are found exclusively in the larval midgut [35]. This protein binds to peptidoglycan to mediate phagocytosis of apoptotic cells and bacteria in *Drosophila* [30, 36]. An integrin from the oyster *Crassostrea gigas* that mediates the phagocytosis toward *Vibrio splendidus* through LPS binding activity is also documented [37]. The thrombospondin-coated apoptotic cells are tethered to the macrophage by CD36, and the vitronectin receptor signals the initiation of phagocytosis (reviewed in [38]). The extracellular matrix protein mindin (spondin-2) is a pattern-recognition molecule binding LPSs and an integrin ligand and is critical for initiating innate immune responses to both bacterial and viral pathogens [28].

Apolipophorin (ApoLp)-III binds to microbial cell wall components, such as gram-negative bacteria LPSs, gram-positive bacteria lipoteichoic acids, and fungal β-1,3-glucans. These binding properties impart to ApoLp-III a role pertaining to the pathogen recognition receptor (PRR) (reviewed in [39]). Thaumatin-like proteins (TLPs) can bind to β-1,3-glucans [40], indicating a potential function in pattern recognition in insect immunity. From the droplets, both ApoLp and TLP were detected (Additional file 1: Table S1; Fig. 2).

### Proteins involved in phenoloxidase activation

Insect peroxinectin plays a role in prostaglandin (PG) biosynthesis [41], and an iPLA2-γ that can promote prostaglandin production has also been identified [42]. This particular phospholipase specifically recognizes the sn-2 acyl bond of phospholipids and catalytically hydrolyzes the bond releasing arachidonic acid and lysophosphatidic acid. Upon downstream modification by cyclooxygenases, arachidonic acid is modified into active compounds called eicosanoids [43]. PGs mediate the release of proPO, which may occur via cell surface GPCRs [44, 45].

A cascade of proteinases triggers activation of the proform of prophenoloxidase-activating enzyme (ppA) into active ppA, which cleaves proPO into active PO and results in melanin production. In insects, venom serine protease (VSP) acts as an arthropod prophenoloxidase-activating factor (PPAF), thereby triggering the phenoloxidase (PO) cascade [46]. The active ppA directly, or through an intermediate proteinase, activates properoxinectin (proPXT), a myeloperoxidase homologue, into peroxinectin (PXT). Serpins negatively regulate both PXT and ppA production. ProPXT lacks the cell adhesion, opsonin and encapsulation-promoting activities of PXT. However, both proPXT and PXT possess peroxidase activity. PXT is associated with an extracellular superoxide dismutase (SOD) and with an integrin. An attractive hypothesis is that an active microbe-PXT-dismutase complex is phagocytosed and results in intracellular killing (insert) through the generation of reactive oxygen intermediates (HOCL) by NADPH oxidase (reviewed in [47]). From the anal droplet, one proPO2, two Serpins (Antitrypsin-like, Spn88Ea), 14 trypsin-like serine proteases (some of them orthologous to H2, P127, P27, P40, P80, P128, H129, P155, and H146 of *Tribolium castanum* [48]; Additional file 1: Table S1, Additional file 3: Table S3), one VSP34-like protein and one VSP-like protein were identified (Additional file 1: Table S1; Fig. 2).

### Proteins involved in oxidative defense

ROS in the gut have a bactericidal role and also act as both intra- and intercellular signaling molecules to induce repair responses or other homeostatic pathways [1, 49]. ROS can be produced within mitochondria or generated by Nox enzymes, and most cellular ROS have a mitochondrial origin [50]. Nox is a protein family composed of seven members. The *Drosophila* genome encodes one Nox5 and one Duox, and the Duox, but not the Nox5, is responsible for the extracellular microbicidal activity in gut mucosal immunity [51]. In *Dendroctonus ponderosae*, two isoforms of Nox5 (XP_019771050.1, XP_019771051.1) and one Duox (XP_019766183.1) are encoded in the genome. From the anal droplet, only one weevil-derived Nox5 was detected. However, seven weevil-derived enzymes involved in scavenging of ROS were detected, including two copper/zinc SODs, two peroxiredoxins (PRX) (reduces H_2_O_2_ in the presence of dithiothreitol [52]), one catalase (CAT), one ferritin (FTH) [53], one transferring (TF) [53] and one Vitellogenin (Vg) [54] (Additional file 1: Table S1; Fig. 2). These results indicated that the excessive ROS was cleared to maintain a balanced defense.

### Insect AMPs from the anal droplet

*Drosophila* AMPs in the gut are apparently required to maintain a healthy gut microbiota [55]. From the anal droplet, only two AMPs were identified. A plant-originated thaumatin-like protein was one of the AMPs, which has antifungal activity against filamentous fungi *Beauveria bassiana* and *Fusarium culmorum* [56] (Additional file 4: Table S4). Thaumatin-like proteins are type 5 pathogenesis-related proteins, which are detected in high levels in plants upon biotic (including insect stress) or abiotic stress [57]. However, in insects, the function of thaumatin-like proteins is not well studied yet. A very recent study showed that in the cereal weevil *Sitophilus oryzae*, the expression of thaumatin drastically decreases during the population burst of midgut endosymbiont *Sodalis pierantonius* [58]. Thus, the discrimination of thaumatin as an AMP might be premature. The other AMP was c-type lysozyme 3 (Additional file 1: Table S1, Additional file 4: Table S4), which is expressed in the gut of *Drosophila* and is suggested to play a role in nutrition by lysis of the microbial cells rather than in immunity (reviewed in [5, 59]). These results are consistent with those in *Drosophila*, i.e., normal gut residents do not incite AMP production [60].

### Anal droplet proteins involved in immune pathways

Two major signaling pathways, Imd and Toll, control the expression of AMPs in the body cavity, but in the case of the midgut, AMP production is induced in response to Imd signaling (reviewed in [1]). In mammalian cells, activation of Tak1 requires an upstream kinase complex consisting of Tak1 and two specific Tak1-binding proteins, Tab1 and Tab2. Tab1 functions as an activator of Tak1, and Tab2 functions as an adaptor protein that links Tak1 to the upstream regulator Traf6 in the IL-1 signaling pathway [61]. The *Drosophila* Imd pathway bifurcates into the JNK pathway at the level of Tak1 and Tab2 (reviewed in [62]). From the anal droplet, proteins Imd, Tab1 and Tab2 were detected (Additional file 1: Table S1, Additional file 2: Table S2; Fig. 2), but Tak1, Ird5 and Relish were not. However, other proteins, for example, Caspar [1], Pgrp-lb [1] and LysM (Lysmd2) [63], which decrease Imd signaling, were also identified from the anal droplet.

Proteins involved in the Toll pathway were also identified from the anal droplet (Additional file 1: Table S1, Additional file 2: Table S2; Fig. 2). Endogenous humoral factors from microbes activate the toll pathway ‍in the hemolymph in which the endogenous ligand ‍Spz‍ is processed to its active form through a ‍proteolytic cascade [64].‍ Persephone (Psh) is an extracellular Clip-domain serine protease activating Spz through a proteolytic cascade that is partly independent of proteoglycan sensing [64]. Other molecules involved in the toll pathway were also detected, including pelle, spz4, and TLRs (TLR7 and one unclassified TLR). However, Myd88 and Tube were not detected from the anal droplet.

NF-kappa-B inhibitor cactus (IκB homolog) was also detected from the anal droplet. Furthermore, a Cactin (Cactus interactor) was identified that negatively regulates NF-kappaB transcription factor activity [65] and inhibits the promoters of *Drosophila* and shrimp AMPs [66]. TmCactin may serve as an important regulator of innate immunity, mediating AMP responses against both gram-positive and gram-negative bacteria in *Tenebrio molitor* [67]. Hun et al. proposed that cactin acts in both *Drosophila* and *T. molitor* as a positive regulator of Toll signaling, whereas in humans, cactin functions as a negative regulator [67]. However, no AMPs were detected from the anal droplet, suggesting cactin functions as a negative regulator in the weevil gut. These results suggest that negative regulators of the Imd pathway repress AMPs in the gut to protect the normal microbiota, thereby maintaining the balance between immune tolerance and immune response (reviewed in [68]).

### Proteins involved in phagocytosis

The entire process of phagocytosis of microorganisms by phagocytes can be divided into a series of defined steps: recognition, attachment, signaling, engulfment and phagosome maturation. Phagocytosis is a special endocytosis, and the phagosome ultimately fuses with a lysosome. From the anal droplet, 11 proteins (four HSPA1s, HGS, RNF41, IQSEC, SH3GL, RAB11FIP1_2_5, DAB2, EHD1, PSD, RABEP1) involved in endocytosis, 12 proteins (uidA, NAGA, uidA, CTSL, CTSB, MAN2B1, LIPA, CTSL, GGA, MAN2B1, E3.2.1.25, SMPD1) involved in lysosome pathways and 11 proteins (four ACTB_G1s, two CTSLs, SEC22, ITGB1, THBS2S, HGS, ATPeV1C) involving the phagosome pathway were identified by KEGG (Additional file 2: Table S2; Fig. 2).

#### Proteins involved in microbe internalization and phagosome formation

In addition to the proteins (F-actin, Sec22) identified by KEGG, other proteins involved in phagosome formation were also found in the anal droplet: Cofilin, Aip1, Carmil1, Ilk and Formin (Additional file 1: Table S1; Fig. 2). Cofilin promotes actin cytoskeletal remodeling to form phagocytic cups by accelerating actin turnover and thereby facilitating phagosome formation [69]. Aip1 increases the filament disassembly activity of Cofilin and restricts Cofilin localization to cortical actin patches [70]. By uncapping actin filaments, mammalian Carmil1 increases actin polymerization and plays a role in endocytosis and phagocytosis [71]. Integrin-linked kinase (ILK) couples integrins and cytoskeletal proteins and is an essential link between integrins and uptake of bacterial pathogens by epithelial cells [72]. Rho-GTPases can induce accumulation of mDia1 (mammalian diaphanous related formin 1) and polymerize actin in the phagocytic cup (reviewed in [31]).

#### Proteins involved in phagosome maturation

In addition to the proteins (vATPase, cathepsin) identified by KEGG, other proteins involved in phagosome maturation were also found in the anal droplet: VPS54, Kinesin, Dynein and Rab GTPases (Additional file 1: Table S1; Fig. 2). The maturation of phagosomes involves interactions with other cellular organelles and includes the stages early phagosome, intermediate phagosome, late phagosome, and phagolysome [30, 31]. GARP (Golgi-associated retrograde protein) complex, functioning in traffic from endosomes to the trans-Golgi network, is composed of VPS51, VPS52, VPS53, and VPS54 [73]. Kinesin is the major microtubule motor involved in trafficking materials required for the early events in phagocytosis [74]. From the anal droplet, two kinesins (Kif23, Kif18a), one trafficking kinesin-binding protein (Milt) and one carboxy-terminal kinesin 2-like protein were detected. Dynein, which travels toward the minus end of microtubules, propels phagosomes centripetally toward the microtubule-organizing center at which late endosomes/lysosomes that are themselves endowed with Rab7, RILP and dynein frequently accumulate [75]. Rab GTPases are at the central node of the machinery that regulates the dynamic process of interactions between phagosomes and intracellular compartments [76]. From the anal droplet, four proteins (Rab-3, Rab-14, Rab-27A, Rab-32) involved in the network of phagosomal Rab GTPases were identified of which Rab-3 and Rab-27A are the less common Rab proteins, whereas Rab-14 and Rab-32 are the most common ones [76].

The final step in the maturation process is the formation of the phagolysosome (pH ~ 4.5). Key cofactors of bacterial housekeeping enzymes are removed from the phagosomal lumen to prevent bacterial growth. ROS and reactive nitrogen species (RNS) attack bacterial DNA, proteins, and lipids to destroy the pathogen [30]. To date, however, no reports show that phagocytic hemocytes in *Drosophila* have a functional phagocytic respiratory burst pathway. Additionally, no reports show whether *Drosophila* epithelial cells are capable of microbial phagocytosis or that *Drosophila* Duox is involved in such a mechanism (for review, see [51]). In vertebrates, phagocytic respiratory burst (PRB) is generated by Nox2 [51], which is not encoded by insect genomes. Thus, in insects, the production of PRB remains to be elucidated.

#### Proteins involved in phagocytosis regulation

Phagocytosis crucially depends on remodeling of the actin cytoskeleton and on membrane dynamics. Distinct Rho GTPases regulate actin polymerization during phagocytosis and actually define several modes of phagocytosis that are phenotypically different (reviewed in [77]). Members of the Rho-GTPases coordinate actin dynamics, functioning as molecular switches that alternate between active (GTP-bound) and inactive (GDP-bound) states [30]. They are activated by guanine nucleotide exchange factors (GEFs) and inactivated by GTPase-activating proteins (GAPs). From the anal droplet, no Rho-GTPases or downstream elements including WASp, Scar/WAVE and Arp2/3 were detected, whereas two GEFs (Arhgef5, Dock9) and three GAPs (Srgap1, Arhgap39, Abr) were detected (Additional file 1: Table S1; Fig. 2). Arhgef5, a member of the Dbl family of Rho GEFs, strongly activates rhoA and plays a crucial role in regulating cytoskeletal remodeling linked to cell migration and invasion [78]. Dock 9 (dedicator of cytokinesis protein 9) belongs to the Zizimin subfamily (Class D) and is a specific GEF for Cdc42 [79]. Both Srgap1 and Arhgap39 impair the ability of cells to ingest IgG-opsonized targets [80, 81]. Srgap1 inhibits Rho A, and Arhgap39 inhibits Rac1 [80, 81], whereas Abr is specific for Rac and Cdc42 [82].

None of the GEFs for Rab were detected from the anal droplet, whereas three GAPs were detected (Tbc1d9, Tbc1d13, SGSM3) (Additional file 1: Table S1; Fig. 2). TBC1 domain family member 9 (Tbc1d9), a rab GAP gene, regulates the late-endosome trafficking pathway [83]. TBC1 domain family member 13 (Tbc1d13) acts as a GTPase-activating protein for Rab35 [84]. Small G protein signaling modulator 3 (SGSM3) is a specific cellular GAP for Rab5 [85].

### Proteins involved in defense against viruses

The RNA silencing endonuclease Argonaute 2 mediates specific antiviral immunity in *Drosophila melanogaster* [86]. Drosophila Dicer-2 has an RNA interference-independent function that modulates Toll immune signaling, which defends against gram-positive bacteria, fungi, and some viruses [87]. Mosquito RNAi is the major innate immune pathway controlling arbovirus infection and transmission [88]. From the anal droplet, both Argonaute-2 and Dicer-2 were identified (Additional file 1: Table S1, Additional file 3: Table S3), suggesting the weevil uses RNAi to manipulate some microbes, particularly viruses.

### Other proteins related to immune function

Four chitinases were detected from the anal droplet (Additional file 1: Table S1). These chitinases were Group IV chitinases, containing a GH18 domain and a CBM14 domain but lacking an STL region. Group IV chitinases apparently have functions in the intestinal system because they are only expressed in different parts of the gut. This feature suggests that they are involved in the degradation of chitinous material either assimilated with food or as part of the PM. Some of these gut-specific chitinases may also have immune functions. Some are suspected to have antifungal activity, as fungal cell walls also consist of chitin. A SWI/SNF-related matrix-associated actin-dependent regulator of chromatin subfamily A containing DEAD/H box 1 homolog (Etl1, also known as CG5899) that showed defensive response to a gram-negative bacterium [89] was identified from the anal droplet (Additional file 1: Table S1).

## Discussion

Gut microbiota are essential for the health of humans and animals. These microbes increase resistance to infection, stimulate mucosal immune defenses, synthesize essential vitamins and promote caloric uptake by hydrolyzing complex carbohydrates [90]. Similar to pathogens, the native gut flora induces immune responses in the insect host [91]. To date, no specific mechanism to recognize commensal bacteria has been identified in *D. melanogaster* [1]. However, obviously, insects evolved regulatory mechanisms that prevent a deleterious induction of the immune response under basal conditions but allow a rapid elimination of microorganisms on pathogenic infection [1]. First, new epithelial cells are constantly generated at the level of intestinal crypts, and a tight balance is maintained between the self-renewal of cells and their elimination that is crucial to homeostasis and epithelium integrity [92]. Our results showed that epithelium renewal occurred in basal conditions, perhaps via the Wingless pathway. Additionally, a mucus layer and adherens junctions provided physical barriers against gut microbes. Second, the insect gut produces AMPs, ROS (by Duox), lysozymes and PO to defend against pathogens [1, 3]. Our results showed that in basal conditions, lysozymes, PO and ROS functioned in the weevil gut, whereas the Imd pathway and AMP production were repressed.

Lysozymes are considered to play a role in nutrition rather than in immune function. The PO cascade is an extracellular pathway in insects that is triggered by certain pathogen-associated molecular patterns and leads to the melanization of pathogens and damaged tissues [3]. Prophenoloxidase activation in hemolymph is required to survive microbial infections in *Drosophila* [93]. The PPOs in insect molting fluids can be activated by a virulence factor from a fungal spore and effectively melanize and inhibit fungal spore germination [94]. However, the activity of POs in the gut environment is poorly understood in arthropods, although PO secretion is demonstrated in the foregut and hindgut (reviewed in [9]). Our study showed that the PPO activating pathway in the gut was the same as that in hemolymph. PO activity contributes to wound healing by forming a scab at the epithelial injury site. The major function of gut POs is supposed as phenol-polymerization [95], which has been demonstrated in insects [96]. However, melanization induced by microbial infection has been observed in the enterocytes of the hindgut of mutant *Drosophila*, which does not require the recruitment of hemocytes [97, 98], suggesting that the weevil gut PO plays a role in immune function.

For gram-negative bacteria, the insect Duox system produces ROS, which in turn sterilize infected microbes [99]. In the intestine of *Drosophila*, the production of ROS was not observed after gram-positive bacteria *S. aureus* ingestion [99]. Apparently only pathogenic microbes secrete uracil that would activate the Duox pathway [99]. Our study showed that in basal conditions, AMP production was most likely not induced, whereas ROS might be released, which is consistent with reports in *Drosophila* [100]. These results suggest that in basal conditions, gut microbiota include pathogenic microbes (perhaps few in number). Additionally, the animals remained healthy because their defensive system cleared pathogens. Previous studies also show that the Imd pathway does not affect ROS-dependent gut immunity, and ROS-resistant bacteria remained controlled by local AMP expression [99], indicating that ROS are the primary defense, whereas AMPs are secondary. Although Duox was not found in the anal droplet, ROS were supposed in the gut. Antioxidant enzymes including SOD, CAT and TPX1 and nonenzymatic iron-binding proteins including ferritin, transferrin and vitellogenin were all detected, suggesting excessive ROS were cleared. Additionally, the titer of the microbial community associated with increased Imd pathway activation increased during aging of a fly [101], also suggesting that AMP production can be shut down in basal conditions.

From the anal droplet, enzymes involved in the Toll signal pathway were detected, indicating the same activating pathway as that in hemolymph. The Toll pathway is considered to be nonfunctional in midgut AMP production [1], although it is present in the foregut and hindgut (reviewed in [102]), and epithelial Toll-like receptors (TLRs) are activated by symbiotic bacteria in the gut in response to certain pathogens such as *Salmonella enterica* (reviewed in [7]). Very recently, the role of the Toll pathway in *D. melanogaster* gut was demonstrated to be required and sufficient to survive oral infection with *Drosophila* C virus (DCV) [103]. In humans, functions of Toll receptors are supposed to be related to metabolism [104]. These results indicate that the function of the gut Toll pathway is not clear and requires further study.

In humans and mammals, bacteria that do manage to cross the intestinal epithelial cell barrier are phagocytosed by macrophages in the lamina propria. Whereas in the insect gut, cells able to phagocytose bacteria and dying cells are only observed in the *Drosophila* proventriculus (PV) [8]. Our results provide support for this observation. From the anal droplet, many proteins involved in the processes of microbe recognition, internalization, and phagosome maturation and regulation were detected, suggesting phagocytes, perhaps nonprofessional phagocytes, were in the weevil gut. The signaling cascades in phagocytic internalization are known in great detail for the Fc receptors and the complement receptors (e.g., integrin CR3). However, none of the Fc receptors were detected from the anal droplet. Based on the proteins from the anal droplet, we propose that an integrin βν-mediated pathway might be involved in gut phagocytosis, i.e., integrin βν activates Rho by an unknown mechanism, and Rho induces accumulation of mDia, which then promotes actin polymerization [31, 105] (Fig. 2). Although none of the Rho-GTPases were identified from the anal droplet, both GEFs and GAPs for Rho were identified, suggesting Rho was in the gut. Phagocytosis is effective in eliminating bacteria. When phagocytosis is blocked, a systemic immune response ensues (reviewed in [7]). In basal conditions, when phagocytosis is sufficient for a few pathogenic microbes, activation of the systemic immune response (for example, AMPs) is not required. This property might be the reason why phagocytosis is presented, whereas the Imd pathway is repressed in the gut of the weevil.

Insects express high levels of ferritin messages in the hind end of the midgut and the yellow region of the Malpighian tubules that associate primarily with the vacuolar system and serve as iron transporters. The opposite is observed for the mammalian ferritins, which are primarily cytoplasmic and serve as iron storage proteins (reviewed in [106, 107]). The anal droplet ferritins had two possible origins: sloughed cells and/or secreted from the gut. In most insects, intracellular holoferritin is rarely observed in the cytosol or the nuclear compartment but is consistently observed in the secretory pathway [106]. Although the proteomic analysis in this study did not distinguish holoferritin and apoferritin, *Calpodes* larvae secret holoferritin into the posterior midgut lumen, presumably for excretion in the feces [106], suggesting a mechanism for combating iron overload [108]. The exocytosis of iron-loaded ferritin by the midgut is most likely the primary route for iron excretion by insects [109]. Thus, gut ferritin might not be involved in immune defense.

The expression of insect transferrin is up regulated to levels that are similar to those of the antimicrobial peptides after bacterial challenge, and transferrin shows antimicrobial activity (reviewed in [110]). Using immunoblot analysis, the midgut fluid transferrin of *Manduca sexta* has a similar concentration to that of transferrin in hemolymph [111]. However, no reports identify whether the transferrin is holo- or apo-transferrin, which determines whether it has an antibacterial role. Excess iron in the diet down regulates the transferrin, which is rationalized as a failure of the iron withholding strategy [108]. Thus, gut transferrrin may not have an antibacterial role but function as an antioxidant in stress responses such as heat shock, fungal challenge, and H_2_O_2_ exposure [112].

## Conclusions

Our results demonstrated that insect anal droplets contain diverse proteins related to gut homeostasis, suggesting that droplets can be used as materials to investigate the defense of a host against gut microorganisms. Compared with materials such as the gut and frass, anal droplets also have many advantages: animals are not killed, DNA or proteins do not have to be extracted, samples are easily collected, repeated measurement experiments can be designed, etc.

## Additional files

Additional file 1: **Table S1.** Identified proteins by rtandem package and BlastP. Proteins identified by LC-MS were annotated by BLASTP against proteome of *Dendroctonus ponderosae* and NCBI CDD database. This table contains the results of rtandem and the outputs of two local BLASTPs. (XLS 59 kb)

Additional file 2: **Table S2.** KEGG enrichment analysis based on the homologous genes of *Dendroctonus ponderosae.* KEGG enrichment analysis was used to annotate identified proteins by LC-MS. The table only contains those pathways related to innate immunity. (XLS 16 kb)

Additional file 3: **Table S3.** Results of blastP against *Tribolium castanum* proteins presented in ref. [48]. To annotate the identified proteins, BLASTP was carried out against immune proteins of *Tribolium castanum* presented in ref. [48]. This file contains the standard outputs of a local BLASTP. Names of putative *Drosophila melanogaster* orthologs are also presented. (XLS 22 kb)

Additional file 4: **Table S4.** Results of blastP against Antimicrobial Peptide Database (APD). This table shows a standard output of a local BLASTP against Antimicrobial Peptide Database. (XLS 15 kb)

## Abbreviations

AMPs: Antimicrobial proteins
ApoLp: Apolipophorins
CAT: Catalase
CTLs: C-type lectins
DAP: *meso*-diaminopimelic acid
Egfr: Epidermal growth factor receptor
FTH: Ferritin
GAPs: GTPase-activating proteins
GEFs: Guanine nucleotide exchange factors
IECs: Intestinal epithelial cells
LPS: Lipopolysaccharide
M cells: Microfold cells
PGN: Peptidoglycan
PGRPs: Peptidoglycan recognition proteins
PO: Phenoloxidase
ppA: Prophenoloxidase-activating enzyme
PRB: Phagocytic respiratory burst
proPO: Prophenoloxidase
PRX: Peroxiredoxin
PXT: Peroxinectin
RNS: Reactive nitrogen species
ROS: Reactive oxygen species
SOD: Superoxide dismutase
TF: Transferring
TLPs: Thaumatin-like proteins
Vg: Vitellogenin
VSP: Venom serine protease
ZO-1: Zonula occludens-1
βGBP: Beta-1,3-glucan-binding protein
